# Clinical analysis of the acromial height-measuring device combined with new-type clavicular hook plate and standard clavicular hook plate in the treatment of Neer type II distal clavicle fractures

**DOI:** 10.1186/s13018-022-03338-4

**Published:** 2022-10-12

**Authors:** Dashuang Li, Rui Qiao, Na Yang, Kun Zhang, Yangjun Zhu, Zhe Song

**Affiliations:** 1 Department of Orthopaedic Trauma, Hong Hui Hospital, Xi’an Jiaotong University School of Medicine, Xi’an, 710054 Shaanxi China; 2 Xi’an Medical University, Xi’an, 710054 Shaanxi China

**Keywords:** Distal clavicle fracture, Clavicular hook plate, The acromial height-measuring device, Subacromial impingement syndrome, Acromion osteolysis

## Abstract

**Background:**

Distal clavicular fracture is a shoulder joint injury that is common in clinical settings and is generally surgically treated using the clavicular hook plate technique with a confirmed curative effect. However, symptoms, such as shoulder abduction limitation, shoulder discomfort, and postoperative joint pain, may occur in some patients. To overcome these problems, after a previous study we developed an acromial height-measuring device and a new type of clavicular hook plate. This study aimed to investigate whether an acromial height-measuring device combined with an improved new-type clavicular hook plate can better reduce the incidence of complications and improve postoperative function. To provide patients with better treatment effects, an acromion gauge and clavicular hook plate are used.

**Methods:**

A retrospective analysis was performed on 81 patients with distal clavicular fractures admitted to our hospital. They were divided into experimental and control groups according to different plates, and the Constant–Murley score, visual analogue scale score, incidence of acromion osteolysis, and incidence of subacromial impingement syndrome were compared.

**Results:**

Compared with the standard clavicular hook plate, the acromial height-measuring device combined with the new-type clavicular hook plate in the treatment of distal clavicle fractures has a lower incidence of subacromial impingement syndrome with better postoperative functional recovery and quality of life.

**Conclusions:**

We considered the acromial height-measuring device combined with the new clavicular hook plate to be a safe and promising alternative to distal clavicular fractures.

## Background

Clavicle fractures account for approximately 2. 6–5% of all adult fracture types, among which distal clavicle fracture is relatively rare, accounting for approximately 15–25% of all clavicle fracture types [1]. The coracoclavicular ligament is primarily used to maintain stability of the acromioclavicular joint in the vertical direction. According to the relationship between the fracture line and coracoclavicular ligament, the Neer classification divides distal clavicle fractures into three types. Neer II is defined as a fracture occurring in the coracoclavicular ligament with or without a tear. Neer I and III can be treated non-surgically. Neer II has a higher risk of postoperative non-union due to its unstable distal fracture mass and the poor efficacy of conservative treatment. Currently, surgical treatment is required for this condition [2, 3]. There are many internal fixation methods, such as clavicular hook plate, anatomical locking plate, and Kirschner wire fixation, but there is still no optimal treatment plan. The clavicular hook plate can maintain the reduction of the lateral end of the clavicle, and can automatically compress, reduce the movement of the fracture end, and do not affect the rotation of the clavicle. The postoperative effect is accurate and often used for distal clavicular fracture; however, hook plate fixation has a high risk of complications, including acromion osteolysis, SIS, hardware failure and plate displacement, and secondary fractures around the plates [2, 4–7]. Therefore, we retrospectively analysed the data of 32 patients with distal clavicular fracture treated with an acromial height-measuring device combined with improved new-type clavicular hook plate and 49 patients with standard clavicular hook plate in Xi'an Honghui Hospital between June 2018 and June 2020. By comparing the postoperative functional scores and incidence of complications, this study aimed to investigate whether an acromial height-measuring device combined with a new-type clavicular hook plate can better reduce the incidence of complications and improve postoperative function. To provide patients with better treatment effects, the acromial height-measuring device and clavicular hook plate were used.

## Design thought

**Fig. 1:**
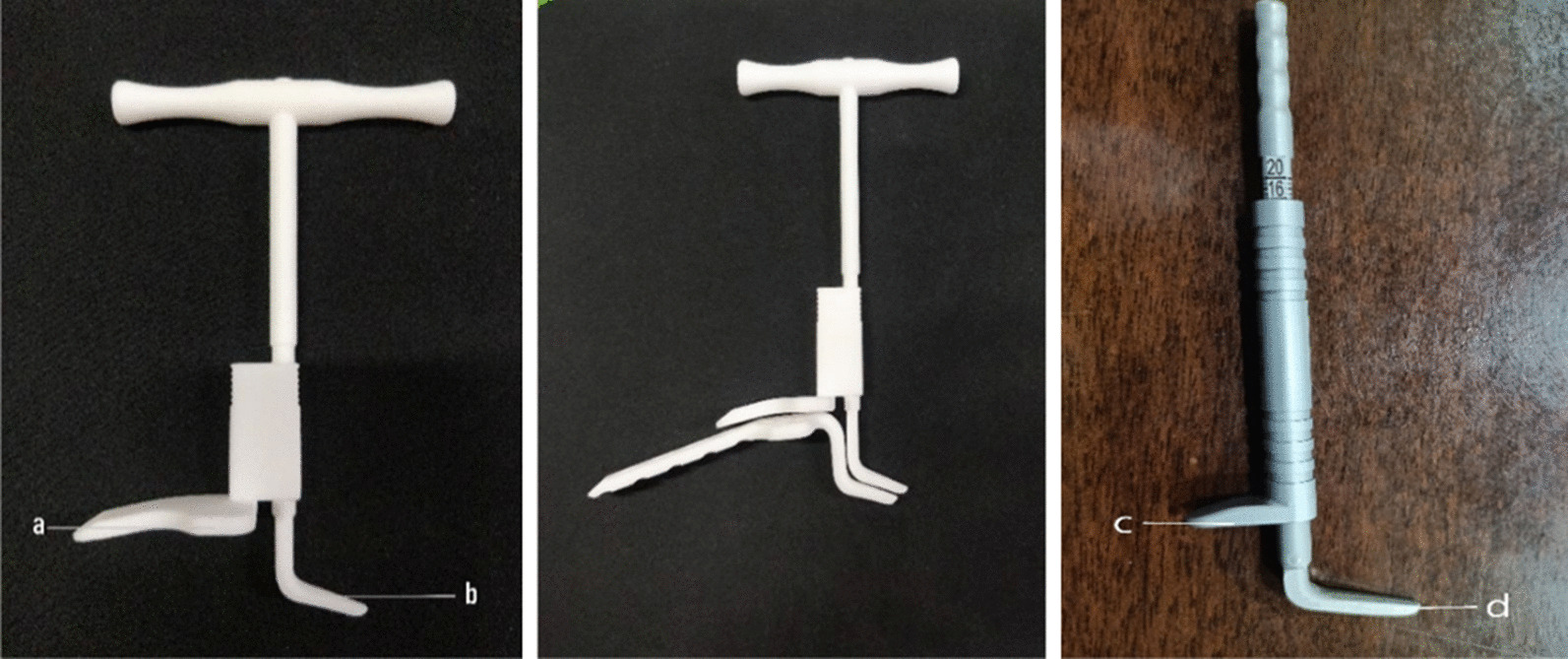
A shoulder height-measuring device developed by our team. The shape of the measuring instrument matches the hook plate currently used. The third picture is a physical object that has been used in clinical practice. During the operation, the c side is placed at the distal end of the clavicle, and the d side is placed under the acromion, and the height of the acromion is measured

**Fig. 2:**
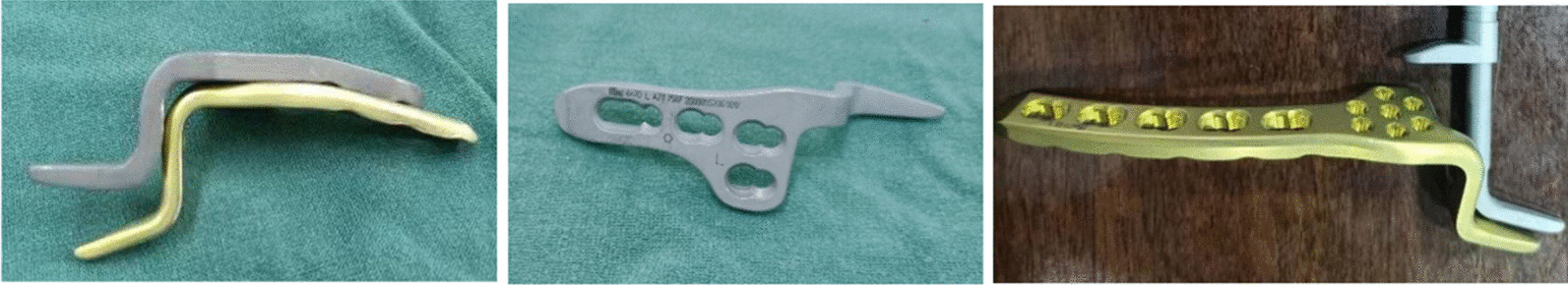
In the past, the end hole of the hook steel plate was a 3.5 mm joint hole, but now it is changed to seven 2.7 mm universal holes. The hook angle of the hook steel plate in the past was 90°, and now a 110° steel plate is added

According to the literature, the emergence of SIS is related to narrowing of the subacromial space [8]. An anatomical study by Amr W. ElMaraghy [9] found that in 89% of the specimens, the hook of the plate passed through the subacromial bursa, 60% of the specimens contacted the supraspinatus tendon, and 60% of the specimens had contact between the plate and acromion concentrated in the hook end. These anatomical findings explain the cause of subacromial bursitis, SIS, and acromion osteolysis. Our team's previous imaging studies on acromial impaction syndrome showed that when the difference between the height of the acromion and depth of the hook plate is > 6 mm, the hook part of the plate does not fit closely with the acromion, and there is too much encroachment on the subacromial space [10]. During exercise, the hook part of the plate and soft tissue of the subacromion squeeze and rub each other, resulting in SIS after the operation. Therefore, the difference between acromial height and hook plate depth was controlled to within 6 mm during the operation in this study. According to the study, we designed the acromial height measurement device (Fig. 1) and improved the clavicular hook plate (Fig. 2). In the past, the hook depth of the standard hook plate was only 12, 15, and 18 mm, and the hook angle was 90°. To make the selected plate more matching, we added 14 and 16 mm depth plates to the new plate to help the selection. And the 110° angle option has been added to all depth plates. An improved hook plate was used during the operation. Distal clavicular fractures are more common in older patients with osteoporosis, and clavicular fractures are more common in older women than in men. Andersen found that the fractures in osteoporosis patients were mostly comminuted, and the fracture pieces were usually small, which was difficult to fix with ordinary screws. The screw holes on the lateral side of the locking plate could achieve multi-directional locking, ensure sufficient stability, and achieve firmer fixation in osteoporosis patients [11]. A study by Chen et al. on the maximum bone density and cortical distribution area of the distal clavicle also provided theoretical support for the location and direction of screw placement [12]. The end of the traditional hook plate was 3.5 mm hole position. The screw may pass through the fracture line in some distal clavicular fractures with small fracture pieces. Therefore, we changed it to seven universal spaces (2.7 mm to facilitate firm fixation of the distal bone pieces in all directions (Fig. 2).

## Patients and methods

### General information

Inclusion criteria: (1) patients with distal clavicle fracture treated with two kinds of clavicular hook plates; (2) patients aged 18 to < 60 years; (3) patients with fresh closed distal clavicle fracture within two weeks after injury; (4) patients with follow-up > 1 year; (5) no clavicle fracture or shoulder-related history; and 6) patients with good compliance. Exclusion criteria were: (1) patients with fractures in other parts of the body; (2) patients with osteoporosis; (3) patients with pathological fracture; and (4) patients who cannot perform functional exercise.

A total of 81 patients with Neer type II fracture who met the inclusion and exclusion criteria and underwent surgical treatment in the Xi'an Honghui Hospital between June 2018 and June 2020 were enrolled in this study. They were divided into two groups according to the plates used: the experimental group (32 patients: 19 males and 13 females; mean age, 39.12 ± 10.63 years). The control group included 49 patients (36 males and 13 females; mean age, 36.78 ± 8.43 years).

### Surgical methods

**Fig. 3:**
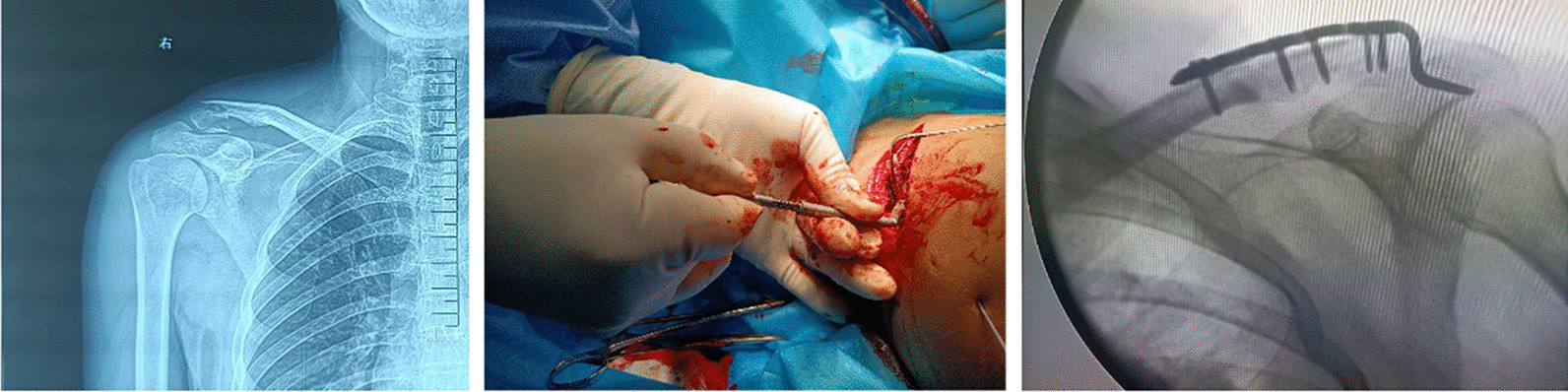
The patient's preoperative X-ray showed that the bone continuity of the right distal clavicle was interrupted; we chose to use the new-type hook plate fixation, which avoided the fracture line well and made the fixation firm

In this study, all the patients under general anaesthesia or nerve block anaesthesia took “beach chair” positions. All the patients in the experimental and control groups were given the clavicular hook plate made by Tianjin Zhengtian Company. All the patients had a rigorous preoperative plan. Elevation of the injured shoulder during surgery and standard anterosuperior approach to the distal clavicle were performed in all the patients. The skin, subcutaneous fat, and fascia layer were cut in turn, and the fractured ends of the clavicle and acromioclavicular joint were examined to protect the periosteum, carefully clean up the blood from the fractured end, reduce and temporarily fix it, and use an acromion metre to measure the acromial height. The right clavicular hook plate was chosen (the difference between the hook portion of the hook plate depth and acromial depth was controlled within 6 mm, and the hook plate with appropriate angle was selected), and the plate was fixed with screws (Fig. 3). The control group did not use an acromial height-measuring device. The distal clavicular fracture was satisfactorily reduced by radiography. Shoulder joint motion was assessed to prevent the hook plate from impinging on the humeral head during abduction or external rotation of the shoulder joint. The wound was closed layer-by-layer and treated with sterile dressing.

### Postoperative management

After the surgery, the upper limb sling was suspended and fixed for four weeks to avoid weight bearing. On the third day after surgery, the affected shoulder was assisted to perform active and passive activities within the pain tolerance range. If the fracture was well fixed, 4–6 weeks later, under the guidance of a professional doctor, shoulder abduction movement was assisted and gradually transitioned to active exercises, such as pendulum movement, passive shoulder flexion, external rotation, and internal rotation to the chest and abdomen. Muscle strength rehabilitation exercises were started. Three months after the surgery, daily activities were resumed according to the patient's condition. Three months later, intensive training was conducted to enhance muscle strength.

### Efficacy evaluation

Acromion osteolysis can be determined if the following symptoms occur: (1) hardening zone appears at the edge of the hook portion of the hook plate; (2) bright shadows appear around the hook portion of the hook plate; (3) the hook portion of the hook plate was cut into the acromion bone; and (4) acromial fracture caused by plate cutting. For SIS, Nikolaus’ diagnostic criteria were adopted: (1) positive shoulder tenderness; (2) When the patient performed upper limb abduction, he experienced pain; (3) pain during active shoulder activity was more obvious than that during passive shoulder activity; and (4) Neer signs were positive. (5) Imaging can be used to detect osteophyte or rotator cuff injuries. We diagnosed the patient as having SIS if three of the above five items appeared.

Sex, age, injury side, time from injury to surgery, and time of postoperative fracture healing were recorded. Regular follow-up was conducted to record fracture healing, Constant–Murley score of the shoulder at three months, 6 months, and 1 year postoperatively, and VAS score at 1 week, 3 months, and 6 months postoperatively, as well as the occurrence of postoperative complications, such as acromion osteolysis and SIS.

### Statistical methods

Statistical analysis was performed using the IBM SPSS software (version 25.0). The measurement data were analysed using the Shapiro–Wilk test to determine whether the data were normally distributed. Age and Constant–Murley scores at 3 and 6 months after surgery were in line with normal distribution and homogeneity of variance, represented by ($\overline{\chi }$± s). An independent sample *T* test was used for comparisons between the two groups. The time from injury to surgery, VAS score data, and Constant–Murley score one year after surgery were non-normally distributed, represented by *M* (*Q*1, *Q*3), and a nonparametric test was adopted. The sex, injured side, acromion osteolysis, SIS results, and other counting data of the two groups were compared using the 2 test. Statistical significance was set at *P* < 0.05.

## Results

**Fig. 4:**
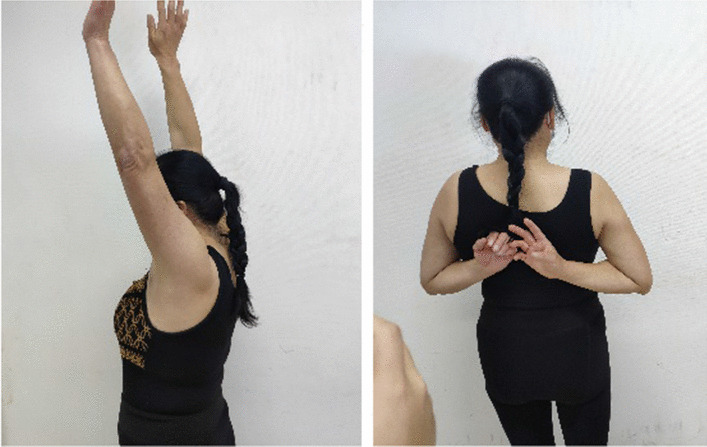
Review 3 months after surgery

**Fig. 5:**
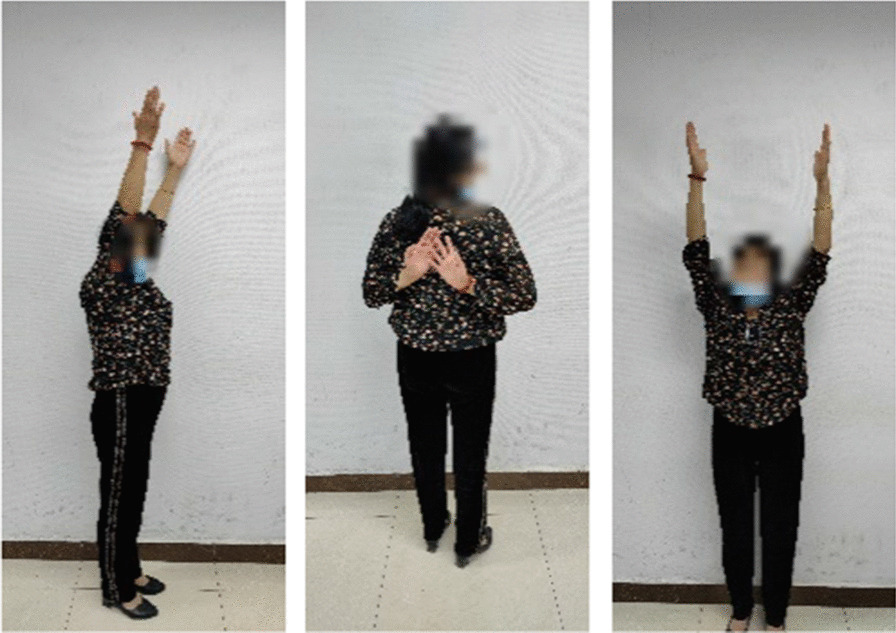
Review 6 months after surgery

**Fig. 6:**
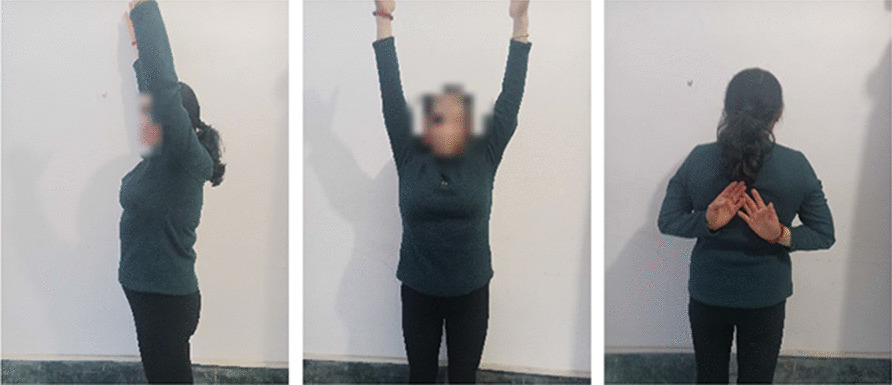
Review 1 year after surgery

Operations were successfully completed in both groups. All the 81 patients were followed-up for 12–22 months with an average of 16.1 months. The healing time was 8–14 weeks with an average of 9.5 weeks. No fracture non-union, delayed union, internal fixation fracture, incision infection, or other complications occurred. There were no statistically significant differences between the experimental and control groups in preoperative general data, such as sex, age, side profile, and time from injury to operation (*P* > 0.05, as shown in Tables 1, 3), indicating comparability. Additionally, SIS occurred in three (9.34%) patients in the experimental group, and in 14 (28.57%) patients in the control group. The SIS in the experimental group was lower than that in the control group, and the difference was statistically significant (*P* < 0.05, see Table 1). The incidence of acromion osteolysis in the experimental group was not significantly lower than that in the control group, and the difference was not statistically significant (*P* > 0.05, see Table 1). Figures 4, 5, 6 is a typical case using the acromial height-measuring device combined with the new-type clavicular hook plate can reduce the incidence of SIS, but has no significant effect on the prevention of acromion osteolysis compared with the standard clavicular hook plate. However, the postoperative functional score in the experimental group was significantly higher than that in the control group. The Constant score of the experimental group at 3 and 6 months after surgery and VAS score at one week and three months after surgery were significantly better than those of the control group (*P* < 0.05, as shown in Tables 2, 3). However, there was no statistically significant difference between the two groups in the Constant score at one year after surgery and VAS score at six months after surgery (*P* > 0.05, as shown in Table 2), indicating that the new-type clavicular hook plate had obvious advantages in patients' functional recovery and quality of life in the short term after surgery, but with an increase in postoperative time, the effects of the two groups were similar.

**Table 1:** Comparison of gender, age, side, acromion osteolysis and SIS between the experimental group and the control group

Group	*n*	Gender		Side		Age	Acromion osteolysis		SIS	
		Male	Female	*L*	*R*		*Y*	*N*	*Y*	*N*
Control group	49	36	13	17	32	39.12 ± 10.63	12	37	14	35
Experimental group	32	19	13	15	17	36.78 ± 8.43	9	23	3	29
Test statistics	–	*Χ*^2^ = 1.764		*χ*^2^ = 1.202		*t* = 1.048	*χ*^2^ = 0.133		*χ*^2^ = 4.302	
*P* value	–	0.184		0.273		0.298	0.715		0.038

**Table 2:** Comparison of constant–Murley score between experimental group and control group

Group	*n*	Constant–Murley score		
		3 months after surgery	6 months after surgery	1 year after surgery
Control group	49	72.22 ± 5.25	84.45 ± 4.00	88.00 (86.00, 90.00)
Experimental group	32	76.22 ± 6.15	86.94 ± 4.28	90.00 (88.00, 92.00)
*t* value	–	*t* = − 3.128	*t* = − 2.662	*Ζ* = − 1.411
*P* value	–	0.002	0.009	0.158

**Table 3:** Comparison of the time from injury to operation and VAS score between the experimental group and the control group

Group	*n*	Injury to surgery time	VAS		
			1 week after surgery	3 months after surgery	6 months after surgery
Control group	49	4.00 (3.00, 5.00)	4.00 (3.00, 4.00)	2.00 (2.00, 3.00)	1.00 (1.00, 2.00)
Experimental group	32	4.00 (3.00, 5.00)	3.00 (3.00, 3.00)	2.00 (1.00, 2.00)	1.00 (1.00, 2.00)
Test statistics	–	− 0.957	− 4.231	− 3.363	− 1.263
*P* value	–	0.338	0.000	0.001	0.207

## Discussion

The surgical efficacy of clavicular hook plates in the treatment of distal clavicle fractures has been widely recognised [13, 14]. The clavicular hook plate can reduce and stabilise distal clavicle fractures through leverage; thus, promoting fracture healing [15]. Compared with other fixation methods, the clavicular hook plate anatomical design is closer to the biomechanical characteristics of the acromioclavicular joint, and can better stabilise the clavicular distal fracture block. At the same time, the acromioclavicular ligament, beak lock ligament, and surrounding soft tissue provide a stable environment without tension, greatly improving the quality of fracture healing and soft tissue repair.

Acromion osteolysis and SIS are common complications of the standard clavicular hook plates. The incidence of SIS after clavicular hook plate surgery is about 19–25% [7, 16]. There are several reasons for this SIS. Soft tissues in the subacromial space, such as supraspinatus tendon, infraspinatus tendon, coracoclavicular ligament, and bursa may be damaged during preoperative trauma, and the insertion of the steel plate at the hook end, scar tissue, and calcification formed during fracture healing also occupy the joint space [9]. The hook portion of the hook plate inserted into the gap under the acromion can lead to a decrease in the shoulder peak. If the hook angle of the hook plate selected during the operation is mismatched or pre-bent poorly, it further reduces the clearance under the shoulder peak. Other studies have shown that elderly patients undergoing hook plate surgery have a higher risk of SIS, which may be related to shoulder tissue degeneration [7, 17]. Macdonald [18] believed that SIS after clavicular hook plate surgery was related to the acromial type, and curved and hooked acromions were more likely to cause SIS than flat acromions. Additionally, the hook portion of the hook plate was fixed under the acromion without screws, and there was fretting in the horizontal direction, which could rub the soft tissue under the acromion to aggravate inflammatory response of the tissue and cause pain, resulting in SIS [19]. ElMaraghy [9] believed that for women, the hook board and tip of the hook board should be pre-bent when necessary, and a hook board with a smaller depth should be selected when necessary. In this study, the incidence of SIS in the experimental group was lower, and the postoperative functional score in the experimental group was higher than that in the control group. This study proved that the acromion height metre combined with the new hook plate can reduce the incidence of SIS. However, the extent to which the difference between the height of the acromion and depth of the hook plate during surgery can minimise the incidence of subacromial impingement syndrome remains to be further studied.

In this study, the incidence of acromion osteolysis in the two groups was not statistically significant. Researchers have continuously explored acromion osteolysis caused by clavicular hook plates. Currently, it is believed that the risk factors for acromion osteolysis mainly include the stress of the hook plate on the acromion, micromotion of the acromioclavicular joint, long duration of internal fixation, and difference in acromial morphology [9, 20, 21]. According to Anshuman [22], 62.5% of patients treated with hook plates required removal of the internal fixation due to shoulder joint irritation or SIS. Due to loss of the coracoclavicular ligament, the proximal fracture is shifted backwards and upwards by the sternocleidomastoid muscle, while the distal bone is shifted inwards and downwards due to upper limb gravity. A clavicular hook plate is used to treat distal clavicular fractures using the lever principle. When the clavicular hook plate is used for fixation, this stress is transferred to the hook portion of the hook plate and acromion [14, 23]. The contact area between the acromion and hook portion of the hook plate determines the pressure between the acromion and hook. The smaller the contact area, the greater the pressure, and the higher the risk of osteolysis, osteotomy, and acromion fractures [24]. Other studies have observed the reaction force of the acromion on the clavicular hook plate and found that the larger the hook angle implanted into the clavicular hook plate, the greater the force on the acromion, and a larger hook angle makes the contact position between the steel plate hook and acromion. Under the same stress through leverage, the shorter the moment arm, the greater the load on the acromion [25]. The lower surface of the human acromion is irregular; however, the shape of the hook portion of the hook plate is different. It cannot be well attached to the inclined surface, but forms point contact, which constantly rubs against the lower surface of the acromion during the movement of the shoulder joint and may cause acromion osteolysis.

Although this study showed that the acromial height measurement device combined with the new clavicle hook plate had less shoulder pain, better functional score, lower incidence of SIS, and obvious advantages over the traditional method, the complications of the hook plate were still not completely resolved. Some scholars believe that early removal of the internal fixation after fracture healing can reduce the incidence of acromion osteolysis [26]. In this study, the hook plates were removed 12 months after confirming good fracture healing. Currently, clavicular locking plates are widely used in the treatment of distal clavicle fractures with relatively fewer complications [27]. However, some scholars believe that the effect of locking plate fixation is not ideal for small fracture pieces, especially for distal clavicle comminuted fractures caused by traction and trauma of the coracoclavicular ligament or other reasons or elderly osteoporotic fractures, and hook plates are very suitable for such fractures [5, 15]. The authors believe that the clavicular hook plate can be selected according to the location of the clavicular fracture line, the size and degree of comminution of the fracture fragment, and whether the fracture is osteoporotic. Hook plates can provide more reliable fixation in patients with relatively small distal fractures, heavily comminuted fractures, and some loose fractures.

This study has several limitations. First, the influence of different acromion types was not considered. Second, in this study, the difference between the height of the acromion and depth of the hook plate was controlled to be < 6 mm, which was a clinical study based on the previous imaging study of our team, but the results of the imaging study were different from the actual intraoperative measurement results and hook plate. The difference between the depth and height of the acromion was not as small as possible. If the difference is too small, it may cause stress fracture of the clavicle after surgery. The extent to which the difference between the height of the acromion and depth of the hook plate during surgery can minimise the incidence of subacromial impingement syndrome remains to be further studied. Third, the sample size of the study was small, and there was a lack of other case investigations in multiple centres and hospitals. A large sample size is required for further studies. Fourth, in this study, the traditional plate group belonged to the early surgical group, while the acromion measurement combined with the new plate group belonged to the late surgical group. During this period, the accumulation of clinical experience and updating of surgical concepts also had a certain influence on the results of the study.

## Conclusions

In the treatment of distal clavicular fractures, the acromial height-measuring device combined with the new-type hook plate has obvious advantages over the standard hook plate in functional rehabilitation and quality of life of patients. Moreover, it can provide sufficient biomechanical strength and is worthy of promotion.

## Abbreviation

SIS: Subacromial impingement syndrome
